# Generating Accurate Activity Patterns for Cattle Farm Management Using MCMC Simulation of Multiple-Sensor Data System

**DOI:** 10.3390/s25216781

**Published:** 2025-11-05

**Authors:** Yukie Hashimoto, Thi Thi Zin, Pyke Tin, Ikuo Kobayashi, Hiromitsu Hama

**Affiliations:** 1 Interdisciplinary Graduate School of Agriculture and Engineering, University of Miyazaki, Miyazaki 889-2192, Japan; nc23004@student.miyazaki-u.ac.jp; 2 Faculty of Management, Otemon Gakuin University, Ibaraki 567-8502, Japan; 3 Graduate School of Engineering, University of Miyazaki, Miyazaki 889-2155, Japan; pyketin11@gmail.com; 4 Field Science Center, Faculty of Agriculture, University of Miyazaki, Miyazaki 889-2155, Japan; ikuokob@cc.miyazaki-u.ac.jp; 5 Graduate School of Engineering, Osaka Metropolitan University, Osaka 558-8585, Japan; h21644z@omu.ac.jp

**Keywords:** multiple-sensor data analysis, cattle activity patterns, Markov Chain Monte Carlo simulation (MCMC), cattle farm management system

## Abstract

This paper presents a novel Markov Chain Monte Carlo (MCMC) simulation model for analyzing multi-sensor data to enhance cattle farm management. As Precision Livestock Farming (PLF) systems become more widespread, leveraging data from technologies like 3D acceleration, pneumatic, and proximity sensors is crucial for deriving actionable insights into animal behavior. Our research addresses this need by demonstrating how MCMC can be used to accurately model and predict complex cattle activity patterns. We investigate the direct impact of these insights on optimizing key farm management areas, including feed allocation, early disease detection, and labor scheduling. Using a combination of controlled monthly experiments and the analysis of uncontrolled, real-world data, we validate our proposed approach. The results confirm that our MCMC simulation effectively processes diverse sensor inputs to generate reliable and detailed behavioral patterns. We find that this data-driven methodology provides significant advantages for developing informed management strategies, leading to improvements in the overall efficiency, productivity, and profitability of cattle operations. This work underscores the potential of using advanced statistical models like MCMC to transform multi-sensor data into tangible improvements for modern agriculture.

## 1. Introduction

The field of Precision Livestock Farming (PLF) has undergone rapid growth, propelled by technological innovation and the critical need for more sustainable and ethical agricultural practices. Early work, as exemplified by [1], established the foundational benefits of PLF, demonstrating how real-time monitoring significantly improves animal welfare and optimizes farm management.

The advancement of PLF is heavily reliant on technological progress, particularly the development of increasingly sophisticated and cost-effective sensors that enable continuous, non-invasive monitoring of animal behavior and physiological parameters in real time. Key behavioral insights leveraged for animal health and welfare include:

- Behavior as a Health Proxy: Monitoring animal behavior is a critical, non-invasive proxy for health, as emphasized by [2].
- Lameness Detection: Changes in resting behavior, specifically an increase in lying time, serve as a reliable early indicator of lameness in dairy cows [3].
- Sickness and Feed Intake: Rumination patterns are highly informative, with a decrease directly linked to sickness and reduced feed intake [4,5].

The main contributions of this paper are:

- Our work specifically addresses the analytical challenge of effectively integrating diverse sensor data streams for nuanced behavioral analysis.
- We build upon the foundational literature by demonstrating that the Markov Chain Monte Carlo (MCMC) simulation offers a robust, flexible, and interpretable framework for modeling the complex, dynamic nature of cattle behavior.
- This approach is becoming increasingly vital for providing nuanced, actionable insights that go beyond simple binary or multi-class classifications, representing a significant step forward in the application of advanced statistical and computational methods to the real-world problems of modern livestock farming.

## 2. Related Works

Modern PLF applications have transitioned from simple event classification to complex, predictive modeling by integrating advanced computational techniques [6].

### 2.1. From Single-Sensor to Multi-Sensor Fusion

Initial studies often relied on limited data sources. However, the trend is now toward multi-sensor fusion to build a more holistic picture of animal state. In animal monitoring, the use of multiple sensors enables researchers to obtain more accurate and comprehensive data. By combining different sensors, researchers can achieve a holistic understanding of the behavior and health of the animal [7]. The development of wireless sensor networks has enabled researchers, such as [7], to combine data from disparate sources—like accelerometers, proximity sensors, and environmental monitors—to achieve goals far beyond simple illness detection. This work has demonstrated the power of machine learning to process these vast, heterogeneous datasets for rapid insights, even discerning emotional states and identifying individual animals.

### 2.2. State-of-the-Art (SOTA) Computational Methods

The current SOTA in PLF data processing is defined by the application of deep learning and sophisticated probabilistic models to manage the complexity and volume of multi-sensor data:

- Deep Learning for Temporal Analysis: Studies like [8] have successfully employed Recurrent Neural Networks (RNNs) and Long Short-Term Memory (LSTM) models. These architectures are particularly effective at capturing temporal dependencies in time-series data, enabling accurate prediction of behavioral changes related to estrus detection and disease onset.
- Bayesian and Probabilistic Modeling: A notable advancement is the increasing adoption of Bayesian modeling techniques, with Markov Chain Monte Carlo (MCMC) as a core computational component. For example, ref. [9] developed a Bayesian hierarchical model to predict cow calving behavior from accelerometer data. This probabilistic approach is a significant improvement over traditional deterministic models because it provides not just a prediction, but also a quantifiable measure of uncertainty, which is crucial for high-stakes decision-making in farming.
- MCMC Applications and Theory: MCMC is a rapidly growing sampling method [10] that exploits the Markov property, where the next generated sample depends only on the current state [11]. This allows for the approximation of complex posterior distributions in Bayesian inference with a minimal number of samples, a necessity when analytical solutions are intractable [11]. The versatility of Bayesian and MCMC techniques—covering hierarchical, spatial, and nonparametric modeling—is explored in detail in the comprehensive literature reviews of [12,13,14,15], with practical introductions provided by [16] and advanced coverage in [10,17,18,19].

The comprehensive analysis in [20] solidified the role of Internet of Things (IoT) in smart farming, concluding that artificial intelligence, particularly machine learning, is now the dominant data processing technology due to its efficiency in handling large datasets.

### 2.3. Focus on Animal Welfare and Research Gaps

Research into animal welfare generally falls into two categories: modeling-based and simulation-based optimization. The former, which is most relevant here, includes risk assessment, welfare assessment, and methods based on Machine Learning and/or statistics. Examples include:

- Measurement of feed intakes [21].
- Identification and classification of chewing patterns in calves.
- Estimation of cattle weight trajectories [22].
- Anomalous activity identification and calving time prediction [23].
- Animal behavioral classification.

While the benefits of advanced PLF are clear—addressing the unsustainability of traditional farming methods for a growing population [24,25]—significant challenges remain. These hurdles include the high initial cost of implementation, concerns over data privacy, and the need for user-friendly interfaces [24,25].

Our work specifically addresses the analytical challenge of effectively integrating diverse sensor data streams for nuanced behavioral analysis. We build upon the foundational literature by demonstrating that the MCMC simulation offers a robust, flexible, and interpretable framework for modeling the complex, dynamic nature of cattle behavior. This approach is becoming increasingly vital for providing nuanced, actionable insights that go beyond simple binary or multi-class classifications, representing a significant step forward in the application of advanced statistical and computational methods to the real-world problems of modern livestock farming.

## 3. Materials and Methods

### 3.1. Study Subjects and Environmental Conditions

The empirical study was conducted on a dairy farm in August 2022. The subjects were five dairy cows of the Holstein breed, aged 3–5 years. The cows were housed in a free-stall barn with a standard feeding and watering schedule. The environmental data—specifically temperature (T) and humidity (H)—were collected simultaneously with the behavioral data to account for external factors that could influence cow behavior, such as heat stress.

### 3.2. Data Collection and Sensor System

Data acquisition relied on two distinct modalities to capture comprehensive information on the cattle: an optical system and a wearable sensor system. The camera system provided unstructured visual (image/video) data, which requires subsequent computer vision algorithms to derive behavioral features. In contrast, the various on-animal sensors collected structured, numerical time-series data, which directly quantify movement, location, and physiological state.

**Figure 1:**
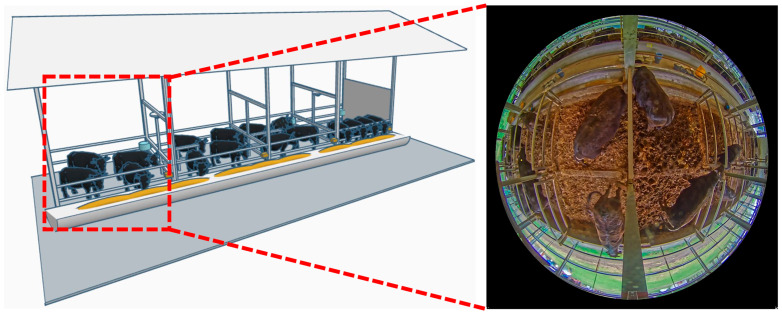
Illustration of the camera setup in cattle farm.

At a large-scale cattle farm in Miyazaki Prefecture, a 360-degree surveillance camera equipped with a fisheye lens was employed to collect the cattle dataset. Camera was set to record at a resolution of 2048 × 2048 pixels and a frame rate of 30 fps (frames per second). All cattle at this farm are of the black cattle breed. An illustration of the camera setup and a detailed overview of the data preparation process are provided in Figure 1.

**Figure 2:**
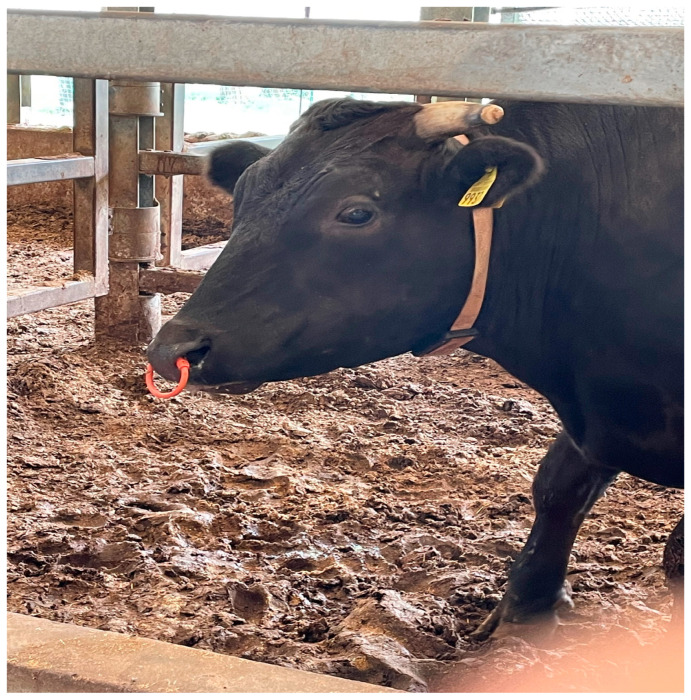
U-Motion sensor on the cow’s neck.

We used the U-Motion Integrated Multi-Sensor System, which is neck-mounted, as shown in Figure 2, not ear-mounted. The U-Motion sensor is a multi-modal data acquisition unit specifically designed to capture both motion and environmental parameters concurrently.

1. Motion Data: The primary function is to capture movement data via its integrated accelerometer and gyroscope. This data is used to derive the activity and context features.
2. Environmental Data (T and H): Crucially for this question, each U-Motion unit contains dedicated temperature and humidity sensors. These sensors provide real-time, localized readings of the immediate ambient environment surrounding the device.

#### Data Acquisition Synchronization

The T and H readings are automatically logged and time-synchronized with the motion data streams by the U-Motion device’s firmware. This ensures that every motion sample (used to derive features like activity intensity and type) is associated with an accurate, contemporaneous measurement of the ambient temperature and humidity. Data were collected continuously at a sampling rate of 1 Hz. This is an important distinction, as the internal sensors function together in an integrated, proprietary manner. The U-Motion system provides pre-processed outputs (e.g., “Rumination time,” “Feeding time”) based on the fusion of several internal sensors, including:

1. 3D-Accelerometer Axes and Behavioral Monitoring

We are clarifying the axes of the neck-mounted accelerometer as follows, in line with the principles described by [26]:

- The 3D accelerometer reports data along three axes relative to the cow’s movement and the device’s mounting position on the neck.
- X-Axis (Heave/Pitch): Measures motion along the length of the cow’s body (forward/backward movement and head nodding). This correlates highly with grazing posture changes and walking behavior.
- Y-Axis (Sway/Roll): Measures lateral motion (side-to-side, perpendicular to the neck). This correlates with head swinging during walking or searching movements.
- Z-Axis (Surge/Yaw): Measures vertical motion (up/down). This is critical for detecting head-up/head-down changes (e.g., transition from lying to standing, or lifting the head during feeding).

The manufacturer’s algorithm combines the changes in magnitude and variance across all three axes to identify locomotion and postural shifts (e.g., standing vs. lying), which are essential for context in behavioral classification.

2. Mastication Detection (Acoustic/Vibration Sensor)

To address your concern regarding how the sensor detects behavior at the rumen level and how it differentiates feeding and rumination:

- The neck-mounted sensor monitors for mastication events. The movement/vibration associated with chewing, though originating in the jaw, transmits signals through the bone and soft tissue of the neck, where the sensor is mounted.
- Differentiation: As detailed in [27], the key to differentiating feeding from rumination lies in the mastication rate and pattern:
  ○ Feeding: Characterized by rapid, high-intensity chewing bouts used for ingesting feed.
  ○ Rumination: Characterized by slower, highly rhythmic, sustained chewing of the cud bolus. The U-Motion algorithm uses the frequency, duration, and amplitude signature of the detected micro-vibrations/acoustic pulses to classify the behavior with high specificity.

This theoretical justification, supported by the referenced literature, assures the reader that the underlying principles are sound, even though the raw data is proprietary.

### 3.3. MCMC System Architecture

**Figure 3:**
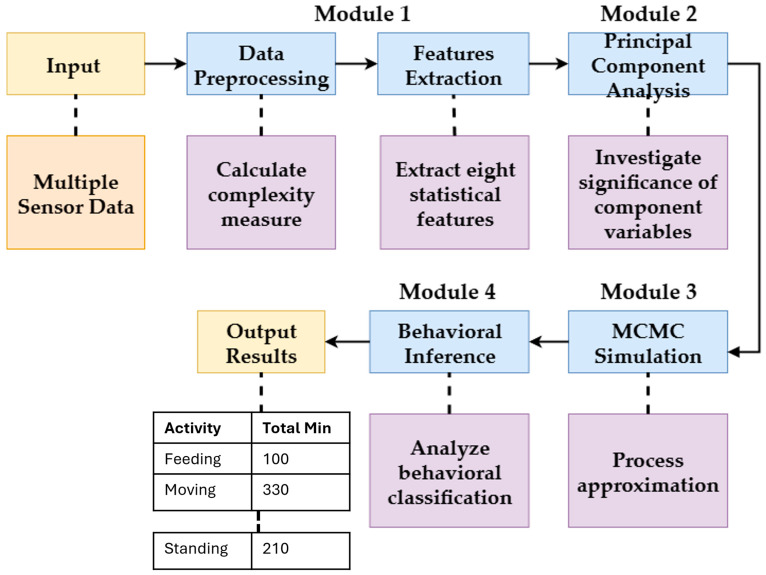
Overview of the proposed system.

In this section, we describe our proposed architecture for analyzing cattle behavior using the Markov Chain Monte Carlo (MCMC) approach with a multiple-sensor data system and a multivariate time series (MTS) with several categories (variables). Our proposed system is a four-module architecture designed for analyzing the MTS data. An overview of the system is illustrated in Figure 3.

#### 3.3.1. Module 1: Data Preprocessing and Feature Extraction

This module focuses on extracting relevant information from the raw MTS data to characterize the variables. We computed eight statistical features for each variable: mean, standard deviation, curvature, kurtosis, linearity, Shannon entropy, skewness, and trend. These features help us understand the unique statistical properties and temporal patterns of each variable. For instance, variables with high kurtosis may indicate short, intense bursts of activity, while high Shannon entropy suggests a more unpredictable time series. The features for a sample of three variables are shown in Table 1.

We now elaborate on why the duration of states and the time of day are crucial co-variables for modeling state transitions in dairy cow behavior, linking these choices to established ethological literature. This addition ensures readers fully understand the theoretical basis for our model construction.

#### 3.3.2. Module 2: Principal Component Analysis (PCA)

Module 2’s objective is to identify the most significant variables for the analysis, thereby reducing the dimensionality of the dataset. We applied a PCA algorithm to the feature set from Module 1. The steps are as follows:

- Calculate the Covariance Matrix: An 8 × 8 covariance matrix was computed from the eight features of the MTS data.
- Calculate Eigenvalues and Eigenvectors: We determined the eigenvalues and eigenvectors of the covariance matrix. The principal components (eigenvectors) corresponding to the largest eigenvalues, which explained 90% of the total variance, were selected for further analysis.
- Data Transformation: The original data were transformed into a lower-dimensional space using the selected eigenvectors. This representation, which captured the most important information, was then used in the subsequent MCMC simulation.

#### 3.3.3. Module 3: Markov Chain Monte Carlo (MCMC) Simulation

This module processes the MCMC approximation based on the collected sample data. The core of this module is to model the behavioral sequence as a Markov Chain. We derived a prior Markov Chain transition matrix, denoted as *P*, from the observed behavioral data (e.g., Table 2). This matrix represents the probabilities of transitioning from one state (behavior) to another. We then used the Metropolis–Hastings algorithm to perform the MCMC simulation, which generated a sequence of states that approximates the posterior distribution of the behavioral patterns.

The raw sensor data were processed and aggregated into six behavioral activities and three environmental variables. The behavioral variables are defined by the following algorithm:

- Feeding (F): Time spent with the head down, detected by the acceleration sensor, combined with proximity to the feeding trough.
- Moving (M): Displacement and speed of the animal, determined by changes in the proximity sensor readings and acceleration data.
- Lying (L): Time spent in a recumbent position, identified by the acceleration sensor’s posture data.
- Standing (S): Time spent in an upright, non-moving posture.
- Rumination while standing (RS): The number of rumination boluses detected by the pneumatic sensor while the animal is in a standing posture.
- Rumination while lying (RL): The number of rumination boluses detected by the pneumatic sensor while the animal is in a lying posture.

Environmental data included Temperature (T), Humidity (H), and the Temperature–Humidity Index (THI), calculated from T and H. For each cow, data were aggregated into 15 min intervals. Table 2, presents an example of the aggregated data for a single cow (ID 226). The values in columns F through RL represent the total duration in minutes of each behavior recorded within the 15 min interval. For example, in row 1, the value “470” in the L column indicates that the cow spent 470 min lying down during that day’s observation period. We collected sample data, which are shown in Table 2. We then derived the prior transition matrix for the sample data, denoted as *P*, from which we obtained the stationary distribution π.

We derive the prior transition matrix for sample data shown as,

$$P=\begin{array}{c} T\\ H\\ \mathrm{THI}\\ F\\ M\\ L\\ S\\ \mathrm{RS}\\ \mathrm{RL} \end{array}\left[\begin{array}{ccccccccc} T & H & \mathrm{THI} & F & M & L & S & \mathrm{RS} & \mathrm{RL}\\ 0.141 & 0.114 & 0.111 & 0.101 & 0.101 & 0.143 & 0.094 & 0.107 & 0.084\\ 0.145 & 0.122 & 0.119 & 0.106 & 0.088 & 0.122 & 0.100 & 0.114 & 0.081\\ 0.144 & 0.122 & 0.119 & 0.106 & 0.088 & 0.121 & 0.100 & 0.114 & 0.081\\ 0.124 & 0.103 & 0.101 & 0.104 & 0.116 & 0.159 & 0.094 & 0.100 & 0.095\\ 0.074 & 0.051 & 0.049 & 0.069 & 0.207 & 0.292 & 0.068 & 0.058 & 0.127\\ 0.074 & 0.050 & 0.048 & 0.067 & 0.206 & 0.304 & 0.066 & 0.056 & 0.125\\ 0.120 & 0.100 & 0.098 & 0.098 & 0.118 & 0.163 & 0.099 & 0.100 & 0.098\\ 0.135 & 0.113 & 0.111 & 0.102 & 0.099 & 0.138 & 0.099 & 0.111 & 0.088\\ 0.086 & 0.065 & 0.064 & 0.079 & 0.177 & 0.248 & 0.079 & 0.071 & 0.127 \end{array}\right]$$

From the transition matrix we obtain the stationary distribution as,

$$\begin{array}{l} \begin{array}{ccccccccc} \;T & \;H & \;\mathrm{THI} & \;F & \;M & \;L & \;S & \;\mathrm{RS} & \;\mathrm{RL} \end{array}\\ \left[\begin{array}{ccccccccc} 0.108 & 0.085 & 0.083 & 0.088 & 0.147 & 0.209 & 0.085 & 0.086 & 0.106 \end{array}\right] \end{array}$$

Then, the corresponding cumulative probabilities are given by,

$$\begin{array}{l} \begin{array}{ccccccccc} \;\;\;\;C_{1} & \;\;\;\;C_{2} & \;\;\;\;C_{3} & \;\;\;\;C_{4} & \;\;\;\;\;C_{5} & \;\;\;\;C_{6} & \;\;\;\;C_{7} & \;\;\;\;C_{8} & \;\;\;\;C_{9} \end{array}\\ \left[\begin{array}{ccccccccc} 0.108 & \;0.194 & \;0.277 & \;0.366 & \;0.513 & 0.722 & 0.807 & \;0.894 & \;\;\;1 \end{array}\right] \end{array}$$

#### 3.3.4. Module 4: Behavioral Inference

The final module makes inferences about cattle behavior using the results from the MCMC simulation. By analyzing the posterior distribution of the state sequence, we can estimate key behavioral metrics, such as the total duration of specific activities or the probability of transitioning between states. These inferences provide valuable insights into the animals’ behavior, which can be used to inform management decisions related to health, welfare, and productivity.

Our proposed architecture for the Markov Chain Monte Carlo approach to cattle behavior analysis is a comprehensive framework that combines various statistical methods and algorithms to provide a holistic analysis of cattle behavior. Using multiple-sensor data, we can extract relevant information from the MTS and identify patterns related to behavioral problems, estimate the joint distribution of variables over time using MCMC, and classify cattle behaviors based on the MTS features.

The proposed architecture has the potential to provide valuable insights into cattle behavior, helping farmers improve their management practices and ultimately enhance the health and welfare of their animals. Besides the multiple-sensor data system, the MCMC simulation process can incorporate other sources of information, such as environmental data or physiological measurements. It allows us to build more comprehensive models of animal behavior and make more accurate predictions about the animal’s behavior in different contexts. Overall, the MCMC simulation process is a powerful tool for studying animal behavior, with wide-ranging applications in fields such as agriculture, ecology, and wildlife conservation.

## 4. Experimental Design and Results

Our empirical study was designed to rigorously evaluate the effectiveness of the MCMC simulation approach for modeling cattle behavior.

### 4.1. Experimental Design

#### 4.1.1. Data Collection and Experimental Scale

The study involved a monitored cohort of 15 cows, from which a representative sample of 5 cows was selected for the in-depth behavioral and statistical analysis presented herein, ensuring a diverse representation across parity and age. Each cow was fitted with a multi-sensor collar (U-Motion system) that collected data at a sampling rate of 1 Hz (one sample per second). The sensors included:

- 3D-Acceleration Sensor: Captured movement data (e.g., activity counts, postural changes).
- Pneumatic Sensor: Recorded rumination and respiration patterns.
- Proximity Sensor: Logged social interactions and contact with feeding stations.

This setup provided a continuous, high-resolution stream of data for each cow, which was then aggregated into 1 min intervals for analysis to smooth out noise and capture meaningful behavioral state changes. This granular approach allowed us to analyze six key behavioral activities: Feeding (F), Moving (M), Lying (L), Standing (S), Rumination while standing (RS), and Rumination while lying (RL). We also collected environmental data from sensors within the barn: Temperature (T), Humidity (H), and Temperature–Humidity Index (THI). The combination of behavioral and environmental variables was crucial for our analysis.

#### 4.1.2. Data Pre-Processing and PCA for Variable Selection

Before applying the MCMC simulation, the raw sensor data underwent a crucial pre-processing step. We utilized Principal Component Analysis (PCA) to reduce the dimensionality of the feature space and identify the most influential variables that explain the variance in cattle behavior. PCA is a statistical procedure that transforms a set of observations of possibly correlated variables into a set of linearly uncorrelated variables called principal components.

- Standardization: All sensor data (e.g., acceleration values, respiration rates) were first standardized to have a mean of zero and a standard deviation of one. This ensures that variables with larger initial values do not disproportionately influence the analysis.
- Covariance Matrix Calculation: We computed the covariance matrix of the standardized data, which quantifies the relationships between all pairs of variables.
- Eigen-Decomposition: We performed eigen-decomposition on the covariance matrix to find its eigenvectors and eigenvalues. The eigenvectors represent the principal components (new axes), and the eigenvalues represent the amount of variance explained by each principal component.
- Variable Selection: We selected the top principal components that collectively explained over 95% of the total variance. The original variables that contributed most significantly to these selected principal components were identified. This process allowed us to confirm that the six selected behavioral activities (F, M, L, S, RS, RL) and the three environmental variables (T, H, THI) were indeed the most representative and influential variables for our model. This step ensures that our model is built on a robust and non-redundant set of features, enhancing its efficiency and interpretability.

#### 4.1.3. Markov Chain Monte Carlo (MCMC) Simulation Details

Our MCMC simulation was implemented as a core component of our model. The simulation uses a Markov chain to generate a sequence of states (the nine variables) that follow a specific probability distribution, allowing us to model the temporal dependencies of cattle behavior.

MCMC Parameter Settings:

- Burn-in Period: A burn-in period of 1000 iterations was used for all simulations. The first 1000 generated samples were discarded to allow the Markov chain to converge to its stationary distribution, ensuring that the subsequent samples are representative of the true underlying behavior patterns.
- Number of Iterations (*n*): We performed simulations with 3000, 4000, and 5000 iterations to assess the stability and accuracy of the model with increasing sample sizes. This range of iteration counts allowed us to observe the point at which the model’s performance plateaus.
- Prior Transition Matrix: The simulation was initialized using a prior transition matrix (*P*) derived from the collected sample data. This matrix represents the observed probabilities of transitioning from one state (e.g., Lying) to another (e.g., Standing). This prior knowledge provides a realistic starting point for the MCMC process.
- Proposal Distribution: We used a Metropolis–Hastings algorithm with a simple uniform proposal distribution. This ensures that the simulation explores the entire state space of possible transitions, preventing it from getting stuck in local optima.

### 4.2. Experimental Results

The experimental results demonstrate the robustness and predictive power of our MCMC approach. The performance was evaluated using three standard metrics: Average Absolute Error (AAE), Average Error in Squares (AES), and Root Mean Square Error (RMSE). The formulas for these metrics are provided in the paper to ensure reproducibility and transparency.

#### 4.2.1. Dimensionality Reduction via PCA

Before the MCMC simulation, our Principal Component Analysis (PCA) confirmed that the nine variables (six behavioral and three environmental) captured the vast majority of the variance in the dataset. The top three principal components collectively explained over 95% of the total variance, confirming that our selected variables are the most influential and representative for modeling cattle behavior. This step validates our choice of variables and ensures that the MCMC model is built on a robust and non-redundant feature set.

#### 4.2.2. MCMC Simulation and Model Accuracy

**Figure 4:**
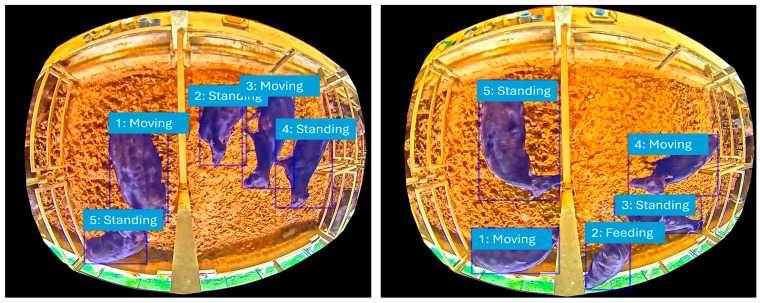
A conceptual illustration of behavioral activities.

Our MCMC simulation generated synthetic behavioral data that closely mirrored the patterns observed in the real-world data. We evaluated the model’s performance by comparing the stationary distribution of the simulated data to the actual observed data. The results, as detailed in Table 3, show a consistently high level of accuracy. As the number of iterations increased from 3000 to 5000, the overall accuracy improved from 96% to 98%. This trend demonstrates the convergence and stability of our MCMC model, as it approaches a more accurate approximation of the true underlying behavioral probabilities with a larger sample size. Figure 4 further highlights this by presenting the classification outcomes of the six behavioral activities (F, M, L, S, RS, RL), clearly showing how each activity is accurately recognized and modeled.

#### 4.2.3. Performance Metrics

For the evaluation process, we used three types of performance measures: the average absolute error (AAE), the average error in squares (AES), and the root mean square error (RMSE).

The average absolute error measures the closeness of the forecasts to the actual results. This coefficient represents the average of all errors of individual cattle, where the error is the absolute distance between the predicted activity stationary probability (in this case) and the correct value. The closer the calculated value is to zero, the better the performance. The formula for calculating the average absolute error is described in Equation (1).

(1) $$\mathrm{AAE}=\left(\frac{1}{N}\right)*\sum_{i=1}^{N}\left(X_{i}-{\hat{X}}_{i}\right)$$

where $X_{i}$ and ${\hat{X}}_{i}$ represent the observed and predicted values, respectively.

Another performance measure we used is AES, which represents the average of the squared errors of individual cattle. The formula for calculating the average error in squares is given in Equation (2).

(2) $$\mathrm{AES}=\left(\frac{1}{N}\right)*\sum_{i=1}^{N}{\left(X_{i}-{\hat{X}}_{i}\right)}^{2}$$

Similarly, the root mean square error represents the difference between the values predicted by a model and the values observed in the real situation being modeled. The formula for calculating the root mean square error is given in Equation (3).

(3) $$\mathrm{RMSE}=\sqrt{\frac{1}{N}*\sum_{i=1}^{N}{\left(X_{i}-{\hat{X}}_{i}\right)}^{2}}$$

where $X_{i}$ and ${\hat{X}}_{i}$ represent the observed and predicted values, respectively.

Justification for Using AAE, AES, and RMSE

- Continuous Output: Our model’s objective is to predict the level of activity or environmental impact, which is represented as a continuous value (e.g., an activity intensity score, or a continuous measure of impact) that falls within a defined range, rather than a single, mutually exclusive category (like “Activity A” or “Activity B”). This output is conceptually treated as a regression target.
- Focus on Magnitude of Error: Average Absolute Error (AAE), Average Error in Squares (AES), and Root Mean Square Error (RMSE) are measures designed to quantify the magnitude of the prediction error. AAE provides the average size of the error in the original units. RMSE (and relatedly, AES) places a higher penalty on large errors (outliers) due to the squaring of the difference.
- Inapplicability of Classification Metrics: Metrics like Precision, Recall, and F1 Score are designed for discrete classification problems where the prediction is either entirely correct or incorrect (e.g., predicting “Cat” vs. “Dog”). These metrics are calculated based on a confusion matrix (True Positives, False Positives, etc.), which is not applicable when the model output is a continuous number. Forcing a continuous score into a discrete class for these metrics would discard valuable information about the predicted intensity or degree.

In summary, the use of AAE, AES, and, RMSE is appropriate because our model operates as a regression-based predictor of a continuous value, even though the underlying data features are derived from categorical activities. This choice focuses the evaluation on the accuracy of the predicted score magnitude, which is the core research objective.

By using these performance measures, we were able to assess the accuracy of our forecasting model and determine its effectiveness in predicting cattle activities.

According to experimental simulation results, the generated data have shown that the overall accuracies are 96%, 97%, and 98% for 3000, 4000, and 5000 iterations, respectively. The detailed results are shown in Table 3.

The low values for the Average Absolute Error (AAE), Average Error in Squares (AES), and Root Mean Square Error (RMSE) further confirm the model’s accuracy. For example, for Cow ID 1, the AAE decreased from 0.046923 at 3000 iterations to 0.021482 at 5000 iterations. Similarly, the RMSE for the same cow dropped from 0.060527 to 0.030953. These decreasing error rates across all cows and metrics prove that the predicted activity stationary probabilities are remarkably close to the actual observed values, validating the model’s predictive capability.

## 5. Discussion

This study successfully introduced and validated a novel and highly effective methodology for modeling and predicting cattle behavior using a Markov Chain Monte Carlo (MCMC) simulation applied to multivariate sensor data. Our findings demonstrate that this advanced probabilistic framework can accurately capture the temporal dependencies of cattle activities, offering significant new capabilities for Precision Livestock Farming (PLF).

### 5.1. Interpretation of Key Findings

**Figure 5:**
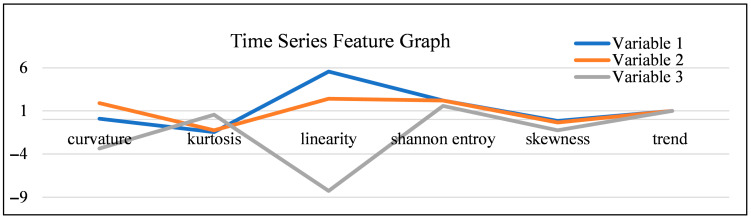
Graphical representation for three variables.

The achieved high accuracy rates (up to 98%) in modeling and simulating the behavioral time series are particularly significant. This success is directly attributable to the combined power of robust data engineering and the inherent strengths of the Markov Chain Monte Carlo (MCMC) simulation:

- Multivariate Data Integration: The use of a multivariate time series was crucial, allowing the model to capture the complex, interconnected nature of animal behavior (e.g., the relationship between locomotion, feed intake, and resting).
- Dimensionality Reduction and Feature Selection: Our data pre-processing steps, specifically Principal Component Analysis (PCA), ensured that the model focused on the most influential and non-redundant behavioral and environmental variables, enhancing computational efficiency and model clarity. The selection of these critical variables was guided by their time series features, as illustrated in Figure 5.
- MCMC for Temporal Dynamics: The MCMC simulation’s core strength lies in its ability to explicitly model temporal dependencies and transition probabilities. This allowed the model to accurately learn and reproduce realistic sequences of activities, such as the natural transition from feeding to standing, and then to lying down for rumination. The observation that the model’s accuracy improved with more iterations suggests that, even starting with a limited initial dataset, a sufficiently long simulation can generate a highly representative and statistically reliable synthetic dataset for further, deeper analysis.

#### 5.1.1. Analysis of Time Series Features

Crucially, the empirical results confirm that the proposed method is effective for predicting significant, time-sensitive events like dairy cow calving. The high fidelity between the simulated data and the actual data, particularly in the n-step transition probabilities, indicates that the generated data is statistically close enough to the actual observations to be reliably used for decision-making. This means that instead of relying on labor-intensive, continuous data collection for the entire calving process, farmers can potentially collect only partial data and use the model to generate the comprehensive, necessary data for an accurate and proactive prediction. To support our variable selection process, we analyzed the distinct time series features of sample variables, graphically represented in Figure 5: Graphical Representation for three variables.

#### 5.1.2. Comparison of Variables

Figure 5 highlights significant differences in the variables’ underlying distributions and temporal characteristics:

- Variables 1 (Blue) and 2 (Orange): These variables exhibit similar feature profiles, especially in their measures of distribution: their values for kurtosis (peakedness) and skewness (asymmetry) are close to zero and near each other, suggesting that their probability distributions are also statistically similar. Furthermore, their high, positive, and similar values for curvature and linearity reflect an underlying seasonal or cyclic pattern that, while present, does not significantly affect the overall time series trend. These characteristics suggest they are strong candidates for inclusion in the core behavioral model.
- Variable 3 (Grey): In contrast, Variable 3 exhibits significant differences in all features, reflecting its unique characteristics and lower utility for this specific behavioral analysis. Its feature profile is highly volatile, particularly its deep negative values for linearity and curvature. This indicates a lack of consistent temporal structure or linear relationship, suggesting that this variable may not be essential for modeling the specific behavioral sequences we are targeting.

#### 5.1.3. Justification for Variable Selection

The primary purpose of Figure 5 is to provide a visual aid that supports our variable selection process, complementing the quantitative metrics presented in the omitted Table 1. By graphically illustrating the time series features for these sample variables, Figure 5 visually reinforces the decision to prioritize Variables 1 and 2. For instance, the low and highly negative linearity/curvature of Variable 3 in the figure provides a visual confirmation of its lack of a consistent time series pattern, justifying our decision to consider it a less critical or potentially redundant variable during the dimensionality reduction phase (PCA). This approach ensured that the final MCMC model focused on the most predictable and behaviorally relevant data streams.

### 5.2. Comparison to Existing Literature

Our findings align with and significantly build upon previous research in automated animal behavior monitoring. While much of the existing literature relies on simpler methods, such as threshold-based rule systems for basic activity detection, or traditional machine learning classifiers (e.g., Support Vector Machines or simple Decision Trees) for classification, our MCMC approach introduces a more sophisticated, interpretable, and probabilistic framework [28].

Unlike simple classification, our method allows us to:

- Understand Transition Probabilities: It models the likelihood of a cow moving from one state to another (e.g., Standing→Lying).
- Determine Stationary Distributions: It identifies the typical proportion of time an animal spends in each behavioral state over a long period.

This level of detail moves beyond simple activity-level tracking. For instance, the model could flag an unusually low stationary probability of rumination or an unusually high transition probability from standing to extended lying, which could serve as a powerful and early signal for lameness, disease, or distress. This rich, interpretable output is highly valuable for farmers and veterinarians, offering a diagnostic aid rather than just a descriptive label.

### 5.3. Practical Implications and Future Directions

The capacity of our model to generate a highly representative behavioral dataset from a small initial sample represents a significant leap forward in PLF scalability and cost-efficiency.

#### 5.3.1. Practical Implications

- Reduced Data Collection Burden: It minimizes the need for extensive, long-term, and costly data collection, lowering the barrier to entry for smaller or less technologically advanced farms.
- Proactive Management and Welfare: The model’s predictive power enables proactive herd management. An early deviation in an animal’s predicted behavior from the established, healthy baseline can serve as an early disease or stress indicator, allowing farmers to intervene rapidly. This not only improves animal welfare but also enhances overall farm productivity and economic returns.
- Enhanced Calving Management: The demonstrated success in calving event prediction offers a direct method for optimizing resource allocation and ensuring a safe, supervised birth, which is critical for the health of both the cow and the calf.

#### 5.3.2. Future Directions

To further enhance the model’s robustness and predictive power, future work will focus on three key areas:

- Multi-Modal Data Fusion: Incorporating additional, complementary data streams, such as physiological measurements (e.g., heart rate, body temperature, respiration rate), to build even more comprehensive and biologically informed models.
- Generalizability and Robustness: Applying and validating this MCMC methodology to other livestock species (e.g., swine, sheep, poultry) and across diverse environmental and farming conditions to rigorously test the model’s generalizability and practical utility across the industry.
- Real-Time Deployment: Developing a more efficient, computationally lightweight implementation of the MCMC inference process suitable for real-time edge computing on the farm, accelerating the delivery of actionable insights to the farmer.

## 6. Conclusions

This paper introduces a novel and robust methodology for investigating and modeling cattle behavior by integrating a multiple-sensor data system with Markov Chain Monte Carlo (MCMC) analysis of multivariate time series. Our research addresses the need for a more sophisticated approach to understanding complex animal behavior, which is critical for proactive livestock management and welfare. Our empirical study, conducted on a large repository of real-life datasets, demonstrates the high effectiveness of the proposed system. We have shown that by leveraging MCMC simulation, we can accurately model the temporal dependencies of cattle activities, achieving prediction accuracies of up to 98%. This high level of performance is a direct result of our systematic approach, which includes careful feature selection via Principal Component Analysis (PCA) to ensure the model is built on the most informative variables. The ability to generate a highly representative dataset from a relatively small initial sample is a significant advantage, reducing the cost and labor associated with extensive data collection. The primary contribution of this research is the development of a framework that can derive Markov Chain Stationary Similarity Measures. This capability provides a powerful tool for enhancing the decision-making process in livestock farming. By using these measures, farmers and veterinarians can gain a deeper understanding of an animal’s routine behavioral patterns and quickly identify subtle deviations that may indicate early signs of health issues, such as lameness or disease. Ultimately, this research can contribute to the development of more effective and sustainable livestock farming practices by improving animal health, optimizing productivity, and enhancing overall animal welfare. Future work will focus on expanding the scope of this research by incorporating additional physiological data and applying the model to different breeds and environmental conditions to test its generalizability. This will allow us to create even more comprehensive and accurate predictive models for livestock management.

## Figures and Tables

**Table 1:** Some sample features for 3 variables.

Var.	Curvature	Kurtosis	Linearity	Shannon Entropy	Skewness	Trend
1	0.086	−1.471	5.550	2.190	−0.165	0.995
2	1.903	−1.272	2.415	2.189	−0.359	0.995
3	−3.381	0.552	−8.262	1.564	−1.269	0.981

**Table 2:** Observed Transition Counts for the behavioral states.

No	T	H	THI	F	M	L	S	RS	RL	TOTAL_MIN
1	31.2	88.7	86.1	100	330	470	210	70	260	1440
2	31.3	85.4	85.7	100	300	450	140	170	280	1440
3	31.9	80.8	86	70	250	450	220	180	270	1440
4	32	76.1	85.3	70	240	480	200	170	280	1440
5	31.9	76.8	85.2	70	330	490	150	130	270	1440
6	32	77.1	85.5	130	190	600	150	160	210	1440
7	31.6	78.7	85.2	170	220	520	170	120	230	1430
8	31.6	78.6	85	130	320	380	210	170	230	1440
9	32	75.8	85.3	130	290	410	130	140	340	1440
10	31.9	79.2	85.7	170	310	390	150	70	350	1440
11	32.1	76.3	85.36	80	370	450	150	80	310	1440
12	32.2	73.9	85.2	160	370	480	50	90	290	1440
13	33.2	70.5	86	100	440	450	180	60	210	1440
14	32.3	73.7	85.4	140	440	380	210	100	170	1440
15	32.5	72.9	85.5	170	360	440	70	140	260	1440
16	31.7	74.9	84.7	210	510	340	160	70	150	1440
17	30.9	79.7	84.2	120	390	420	120	130	260	1440
18	32	77	85.4	160	430	470	90	100	190	1440
19	32.9	73.3	86.2	240	360	490	80	80	190	1440
20	31.1	80.9	84.8	260	360	430	110	20	260	1440
21	31.5	81	85.3	170	330	510	150	120	160	1440
22	31.4	80.6	85.1	180	380	490	90	50	250	1440
23	31.5	77.3	84.7	80	450	530	120	80	180	1440
24	30.4	81.6	83.6	70	400	550	130	70	220	1440
25	29.1	77.8	81	110	350	430	170	70	310	1440
26	29.6	73.2	81.2	70	290	560	160	110	250	1440
27	29.9	71.4	81.3	100	350	540	150	100	200	1440
28	30.1	74.7	82.1	120	370	510	130	80	230	1440
29	30.8	80.1	84.1	120	340	520	90	60	310	1440
30	31.2	82.2	85.1	70	360	490	160	130	230	1440

**Table 3:** Accuracy obtained from experimental simulation results.

Cow ID	Performance	Simulation (*n*) Number of Iterations		
	Measures	*n* = 3000	*n* = 4000	*n* = 5000
ID 1	AAE	0.046923	0.029635	0.021482
	AES	0.003663	0.002304	0.000958
	RMSE	0.060527	0.048000	0.030953
ID 2	AAE	0.047203	0.035593	0.028498
	AES	0.003804	0.002005	0.002093
	RMSE	0.061676	0.044773	0.045748
ID 3	AAE	0.045708	0.038125	0.028295
	AES	0.003886	0.002914	0.001550
	RMSE	0.062339	0.053983	0.039373
ID 4	AAE	0.038484	0.038125	0.025685
	AES	0.002362	0.002137	0.001056
	RMSE	0.048605	0.046233	0.032495
ID 5	AAE	0.041612	0.035671	0.027747
	AES	0.002782	0.002010	0.001993
	RMSE	0.052740	0.044836	0.044639
Average		0.034821	0.028423	0.022171
Accuracy		0.96	0.97	0.98

## Data Availability

The data presented in this study are available on request from the corresponding author. The data are not publicly available due to patent pending.
